# Surgical Outcomes of Robotic Resection for Sigmoid and Rectal Cancer: Analysis of 109 Patients From a Single Center in China

**DOI:** 10.3389/fsurg.2021.696026

**Published:** 2021-08-19

**Authors:** Jianhong Peng, Weihao Li, Jinghua Tang, Yuan Li, Xueying Li, Xiaojun Wu, Zhenhai Lu, Junzhong Lin, Zhizhong Pan

**Affiliations:** State Key Laboratory of Oncology in South China, Department of Colorectal Surgery, Collaborative Innovation Center for Cancer Medicine, Sun Yat-sen University Cancer Center, Guangzhou, China

**Keywords:** sigmoid cancer, rectal cancer, robotic surgery, surgery outcome, oncological outcome, neoadjuvant chemoradiotherapy

## Abstract

**Background:** Robotic colorectal surgery has been increasingly performed in recent years. The safety and feasibility of its application has also been demonstrated worldwide.However, limited studies have presented clinical data for patients with colorectal cancer (CRC) receiving robotic surgery in China. The aim of this study is to present short-term clinical outcomes of robotic surgery and further confirm its safety and feasibility in Chinese CRC patients.

**Methods:** The clinical data of 109 consecutive CRC patients who received robotic surgery at Sun Yat-sen University Cancer Center between June 2016 and May 2019 were retrospectively reviewed. Patient characteristics,tumor traits, treatment details, complications, pathological details, and survival status were evaluated.

**Results:** Among the 109 patients, 35 (32.1%) had sigmoid cancer, and 74 (67.9%) had rectal cancer. Thirty-seven (33.9%) patients underwent neoadjuvant chemoradiotherapy. Ten (9.2%) patients underwent sigmoidectomy, 38 (34.9%) underwent high anterior resection (HAR), 45 (41.3%) underwent low anterior resection (LAR), and 16 (14.7%) underwent abdominoperineal resection (APR). The median surgical procedure time was 270 min (range 120–465 min). Pathologically complete resection was achieved in all patients. There was no postoperative mortality. Complications occurred in 11 (10.1%) patients, including 3 (2.8%) anastomotic leakage, 1 (0.9%) anastomotic bleeding, 1 (0.9%) pelvic hemorrhage, 4 (3.7%) intestinal obstruction, 2 (1.8%) chylous leakage, and 1 (0.9%) delayed wound union. At a median follow-up of 17 months (range 1–37 months), 1 (0.9%) patient developed local recurrence and 5 (4.6%) developed distant metastasis, with one death due to disease progression.

**Conclusions:** Our results suggest that robotic surgery is technically feasible and safe for Chinese CRC patients, especially for rectal cancer patients who received neoadjuvant treatment. A robotic laparoscope with large magnification showed a clear surgical space for pelvic autonomic nerve preservation in cases of mesorectal edema.

## Background

Colorectal cancer (CRC) is the third most common cancer and a leading cause of cancer death worldwide (1), which is an increasingly important obstacle to gains in life expectancy in China (2–4). Despite improvements in the comprehensive treatment and management of CRC patients in recent years, surgery remains the most effective treatment and offers the possibility of a cure for CRC. The quality of surgery is closely associated with oncological outcome. Therefore, a suitable technique for CRC surgery is urgently needed in clinical practice.

Increasing evidence supported by randomized controlled trials demonstrated that laparoscopic surgery was not inferior to open surgery with respect to short-term surgical outcomes and long-term oncological outcomes (5–9), which is becoming the new standard for colorectal cancer treatment. Many advantages of laparoscopic surgery have been reported, including shorter length of stay, smaller scars, and reduced recovery time (10). However, laparoscopic surgery may present some technical drawbacks, such as loss of three-dimensional (3D) view, long instruments that can increase physiological hand tremor, and loss of dexterity. Recently, robot-assisted laparoscopic surgery (RALS) using the Intuitive Surgical® da Vinci™ surgical system (Intuitive Surgical®, Sunnyvale, CA) was developed to facilitate minimally invasive surgery, and this technique provides a stable 3D view and intuitively transfers movements from the handle to the tip of the instrument with tremor filtering to offer enhanced dexterity (11).

Robotic colorectal surgery has been increasingly performed in recent years, and the safety and feasibility have also been confirmed in previous studies (12–14). Limited studies have presented clinical data for patients with CRC receiving robotic surgery in China. The aim of this study is to present short-term surgical and oncological outcomes of robotic surgery and further confirm its safety and feasibility in Chinese patients with sigmoid and rectal cancer.

## Patients and Methods

### Patient Selection

The medical records of 109 consecutive patients were reviewed. All patients were diagnosed with sigmoid colon or rectal cancer and underwent robotic surgery between June 2016 and May 2019 at Sun Yat-sen University Cancer Center (Guangzhou, China). All cases were staged according to the 8^th^ edition American Joint Committee on Cancer (AJCC) staging system. The patients were excluded from robotic approach according to contraindications for robot-assisted colorectal surgery described by expert consensus on robotic surgery for colorectal cancer (2015 edition) (15). In addition, if patients were unwilling to receive robot surgery, we also excluded the cases. The selected case met the following inclusion criteria: (1) histologically confirmed sigmoid colon or rectal adenocarcinoma; (2) underwent robotic curative resection of tumor using the da Vinci Surgical System (Intuitive Surgical Inc., Sunnyvale, CA, USA); and (3) had a complete record of the whole treatment. The patient demographics, tumor characteristics, type of procedure performed, comorbid conditions, operative variables, including operative time, conversion to open, lymph nodes retrieved, estimated blood loss, and blood transfusion, and postoperative variables, including length of stay, and 30-day mortality were carefully reviewed, and oncological outcomes were assessed. The present study was performed according to the ethical standards of the World Medical Association Declaration of Helsinki and was approved by the Institutional Review Board and Independent Ethics Committees of Sun Yat-sen University Cancer Center. The informed consent requirement was waived based on the nature of this retrospective study, in which patient data were kept confidential.

### Surgical Techniques

**Figure 1:**
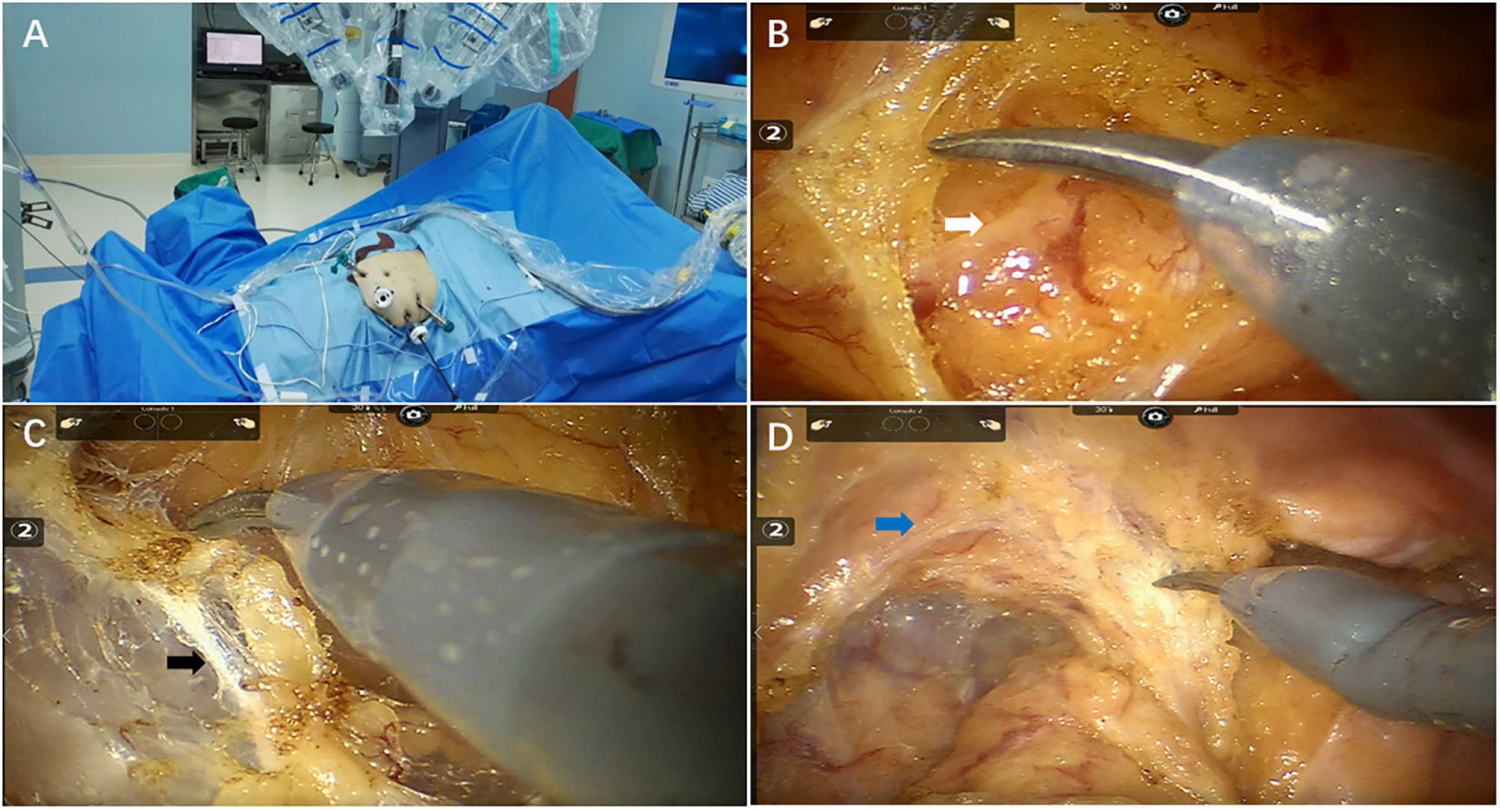
Key techniques of total mesorectal excision after chemoradiotherapy for pelvic autonomic nerve preservation. **(A)** Operation room setup **(B)** Inferior mesenteric nerve (white arrow) preservation **(C)** Hypogastric nerves (black arrow) preservation **(D)** Pelvic plexus (blue arrow) preservation.

In this study, five surgeons performed the all series. The exact trocar placement is shown in Figure 1A. There are 4 trocars placed for the surgery: 1 for the camera, 2 for the robotic arms, and 1 for the assistant. A camera port (12 mm) was placed 3–4 cm above and to the right of the umbilicus. Robotic arm 1 (8 mm) was placed right of the iliac fossa along a line drawn from the umbilicus to the anterior superior iliac spine, one third of the way from the anterior superior iliac spine. Robotic arm 2 (8 mm) was placed 3–4 cm below the xiphoid process. An assistant port was placed (12 mm) at the intersection of the vertical line through McBurney's point and the horizontal line through the camera port.

Total mesorectal excision (TME) and tumor-specific mesorectal excision (TSME) were performed as previously described (15). The procedure of pelvic autonomic nerves preservation (PANP) was performed at the same time (Figures 1B–D). The sigmoid mesocolon was cut along the right pararectal sulcus using the middle approach, and the inferior mesenteric artery was fully exposed. Spleen flexure were released if intestine segment or mesentery is not long enough for anastomosis steps. The inferior mesenteric artery was clamped and cut off approximately 1 cm from the root of the blood vessel in order to protect the superior hypogastric plexus. The “cavity effect” of electric heating equipment was quickly exposed, and Toldt's plane was subsequently entered. The white filamentous connective tissue in Toldt's space was cut sharply using an electric knife and kept in the neurosurgical plane of the white filamentous connective tissue at all times. We separated the posterior wall of the rectum closely behind the fascia propria of the rectum under direct vision in order to protect the inferior hypogastric nerve and the anterior sacral vessel. Similarly, sharp separation of the rectal lateral walls was performed near the outer edge of the rectal ligament and the inside edge of the pelvic plexus to protect the pelvic plexus. The anterior rectal space between the anterior and posterior Denonvilliers' fascia was separated to protect the branches of the pelvic plexus. When the intestine segment or mesentery is not long enough for anastomosis steps, we would conduct splenic flexure taking down.

### Follow-Up

Patients were scheduled for subsequent visits every 3 months for 2 years then semiannually until 3 years after surgery. Physical examination, blood tests for carcinoembryonic antigen (CEA) and carbohydrate antigen 19-9 (CA19-9) levels, abdominal ultrasonography, and chest X-rays were performed every 3 months postoperatively. Chest/abdominal/pelvic computed tomography (CT) and colonoscopy were performed annually. Disease-free survival (DFS) was defined as the interval from surgery to disease recurrence, death, or the last follow-up.Overall survival (OS) was defined as the interval from the date of surgery until death of any cause or the last follow-up. Patients without any event (metastasis or death) at the last follow-up date were regarded as random censoring. The last follow-up visit was in July 2019.

### Statistical Analysis

All statistical analyses were performed using IBM SPSS statistics software, version 21.0 (IBM Corp., Armonk, NY, USA). All of the continuous data are expressed as the means with standard deviation and range. All of the categorical data were calculated as numbers and percentages. The 2-year OS rate and 2-year DFS rate were calculated using the Kaplan-Meier method.

## Results

### Patient Characteristics

**Figure 2:**
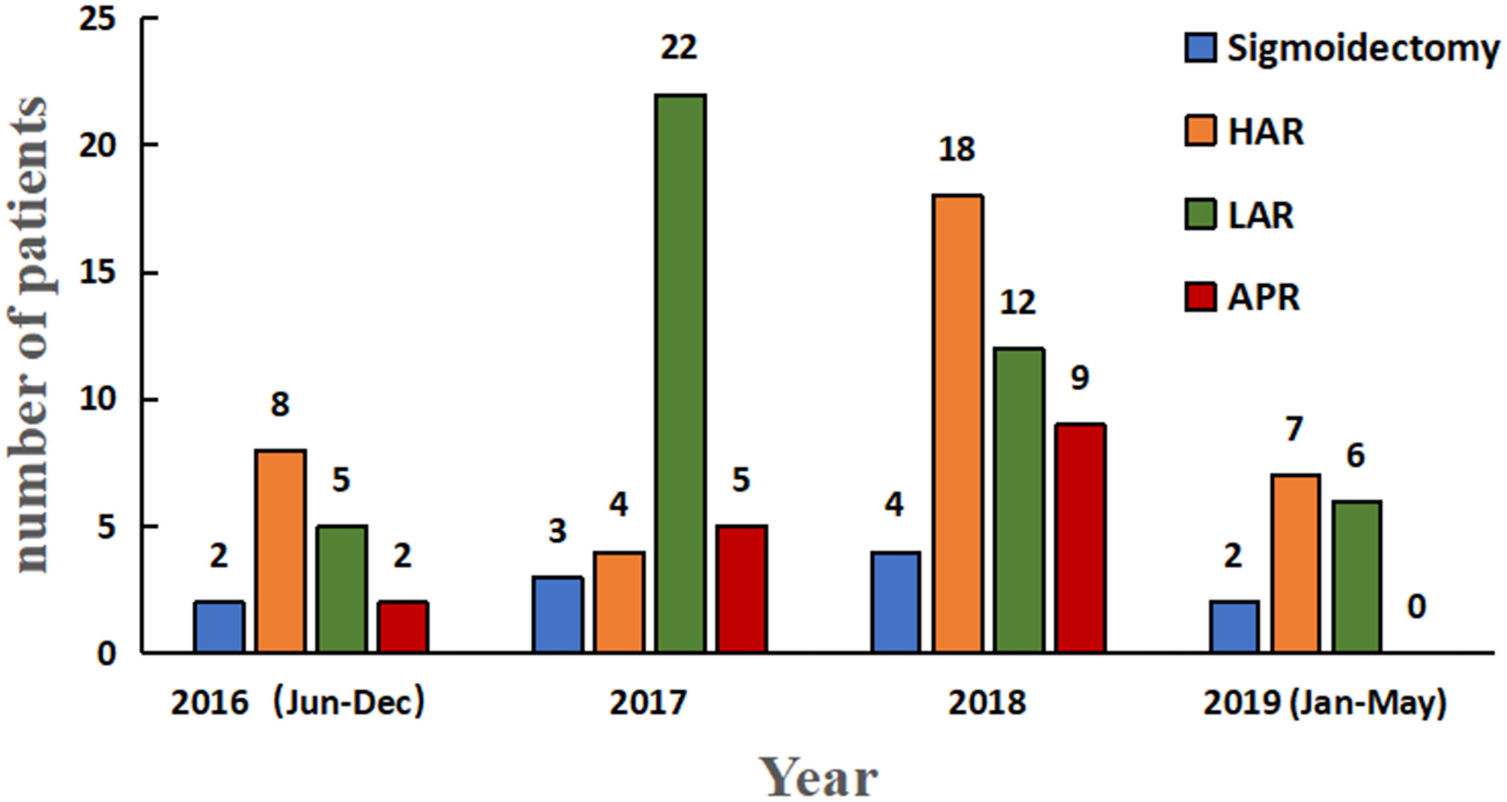
Histogram depicting year-wise distribution of robotic sigmoidectomy. HAR, high anterior resection; LAR, low anterior resection; APR, abdominoperineal resection for colorectal cancer.

Over a 3-year period, 10 (9.2%) patients underwent sigmoidectomy, 38 (34.9%) underwent high anterior resection (HAR), 45 (41.3%) underwent low anterior resection (LAR), and 16 (14.7%) underwent abdominoperineal resection (APR) (Figure 2). Their demographic features and clinicopathological characteristics are summarized in Table 1. Of the total 109 patients, 35 (32.1%) patients presented with sigmoid colon cancer and 74 (67.9%) patients had rectal cancer. Seventy-five patients (68.8%) were males, and 34 (31.2%) were females, with a median age of 59 years (range, 31–82 years). The mean body mass index (BMI) was 22.8 ± 3.0 and comparable between the patients with sigmoid cancer and rectal cancer. Preoperative clinical stage included 17 (15.6%) stage I, 43 (39.4%) stage II, 45 (41.3%) stage III, and 4 (3.7%) stage IV. Ten (9.2%) patients underwent sigmoidectomy. Thirty-eight (34.9%) of the 74 patients with rectal cancer, 37 (50%) received neoadjuvant chemoradiotherapy (CRT) and 4 (5.4%) received neoadjuvant chemotherapy.

**Table 1:** Clinical characteristics of study population.

**Variables**	**Total (***n*** = 109)**	**Sigmoid cancer (***n*** = 35)**	**Rectal cancer (***n*** = 74)**
Age [median (range), years]	59 (31–82)	60 (34–82)	57 (31–73)
Age > 65 years (*n*,%)	30 (27.5)	10 (28.6)	20 (27.0)
Male gender (*n*,%)	75 (68.8)	25 (71.4)	50 (67.6)
BMI (mean ± SD, kg/m^2^)	22.8 ± 3.0	22.7 ± 2.8	22.9 ± 3.2
<18.5	5 (4.6)	1 (2.9)	4 (5.4)
18.5–23.9	63 (57.8)	23 (65.7)	40 (54.1)
24–27.9	36 (33.0)	10 (28.6)	26 (35.1)
≥ 28	5 (4.6)	1 (2.9)	4 (5.4)
ASA classification (*n*,%)			
1	18 (16.5)	5 (14.3)	13 (17.6)
2	84 (77.1)	26 (74.3)	58 (78.4)
3	7 (6.4)	4 (11.4)	3 (4.1)
Smoking history (*n*,%)	23 (21.1)	7 (20.0)	16 (21.6)
Hypertension (*n*,%)	29 (26.6)	11 (31.4)	18 (24.3)
Diabetes mellitus (*n*,%)	14 (12.8)	4 (11.4)	10 (13.5)
Bowel obstruction (*n*,%)	3 (2.8)	2 (5.7)	1 (1.4)
Weight loss within 6 months (*n*,%)	27 (24.8)	10 (28.6)	17 (23.0)
Hemoglobin (mean ± SD, g/dl)	132.2 ± 17.6	133.2 ± 20.2	131.7 ± 16.4
Severe anemia (*n*,%)	2 (1.8)	1 (2.9)	1 (1.4)
Albumin (mean ± SD, g/dl)	41.8 ± 3.6	41.8 ± 4.2	41.9 ± 3.4
Median DAV [median (range), cm]	10 (1–30)	20 (16–30)	7 (1–15)
>15	35 (32.1)	35 (100)	0
11–15	13 (11.9)	0	13 (17.6)
6–10	35 (32.1)	0	35 (47.3)
≤ 5	26 (23.9)	0	26 (35.1)
Preoperative TNM stage (*n*,%)			
I	17 (15.6)	4 (11.4)	13 (17.6)
II	43 (39.4)	15 (42.9)	28 (37.8)
III	45 (41.3)	15 (42.9)	30 (40.5)
IV	4 (3.7)	1 (2.9)	3 (4.1)
Neoadjuvant CRT (*n*,%)	37 (33.9)	0	37 (50.0)
Neoadjuvant chemotherapy (*n*,%)	4 (3.7)	0	4 (5.4)

*BMI, body mass index; ASA, American Society of anesthesiologists; SD, standard deviation; DAV, inferior tumor margin from the anal verge; TNM stage, tumor-node-metastasis classification; CRT, chemoradiotherapy*.

### Intraoperative Outcomes

The intraoperative outcomes are presented in Table 2. The median operative time for robotic surgery was 270 min, with a range of 120 min to 465 min. Median intraoperative transfusion volume for the total cohort was 2,000 ml (range 1,000–4,500 ml). Median intraoperative urine volume for the cohort was 400 ml (range 100–2,100 ml). Median estimated blood loss for the cohort was 50 ml (range 20–400 ml). Three patients had blood transfusion, including two patients in the APR group (12.5%) and one patient in the sigmoidectomy and HAR group (2.1%). None of the cases was converted to an open or laparoscopic procedure, and no intraoperative ureteral injury occurred. Twenty-two patients underwent preventive ileostomy, including four patients in the sigmoidectomy and HAR group (8.3%) and 18 patients in the LAR group (40.0%). Among total patients in this study, there was no case receiving splenic flexure taking down.

**Table 2:** Intraoperative outcomes of total patients.

**Variables**	**Total (***n*** = 109)**	**Sigmoidectomy + HA (***n*** = 48)**	**LAR (***n*** = 45)**	**APR (***n*** = 16)**
Procedure time [median (range), minutes]	270 (120–465)	240 (120–435)	300 (165–450)	295 (170–465)
Intraoperative transfusion volume [median (range), ml]	2,000 (1,000–4,500)	2,000 (1,000–3,500)	2,000 (1,000–4,500)	2,500 (1,500–3,300)
Intraoperative urine volume [median (range), ml]	400 (100–2,100)	400 (100–1,600)	350 (100–2,100)	500 (200–2,000)
Estimated blood loss [median (range), ml]	50 (20–400)	50 (20–300)	50 (50–400)	100 (30–300)
Blood transfusion, *n* (%)	3 (2.8)	1 (2.1)	0	2 (12.5)
Conversion, *n* (%)	0	0	0	0
Ureteral injury, *n* (%)	0	0	0	0
Preventive ileostomy, *n* (%)	22 (20.2)	4 (8.3)	18 (40.0)	0

*HAR, high anterior resection; LAR, low anterior resection; APR, abdominoperineal resection*.

### Pathological Outcomes

The pathological outcomes are presented in Table 3. Pathological stages were stage 0 in 12 patient, stage I in 27 patients, stage II in 38 patients, stage III in 28 patients and stage IV in 4 patients. There were 41 patients with rectal cancer who had received neoadjuvant treatment, and 11 of these patients exhibited a pathologically complete response (pCR). Another one patient who achieved pCR was a sigmoid colon cancer patient who received neoadjuvant chemotherapy. All case received a radical resection and achieved a status of no evidence of disease after surgery.

**Table 3:** Pathologic outcomes.

**Variables**	**Total (***n*** = 109)**	**Sigmoid colon cancer (***n*** = 35)**	**Rectal cancer**	
			**Without neoadjuvant treatment (***n*** = 33)**	**With neoadjuvant treatment (***n*** = 41)**
Tumor size [median (range), cm]	2.5 (0.5–13.0)	4 (10.0–13.0)	2.7 (1.5–6.5)	1.5 (0.5–5.5)
Tumor differentiation, *n* (%)				
No tumor cells	18 (16.5)	4 (11.4)	1 (3.0)	13 (31.7)
Well-differentiated carcinoma	0	0	0	0
Moderate carcinoma	76 (69.7)	28 (80.0)	25 (75.8)	23 (56.1)
Poor carcinoma	14 (12.8)	3 (8.6)	7 (21.2)	4 (9.8)
Mucous carcinoma	1 (0.9)	0	0	1 (2.4)
Pathological T stage, *n* (%)				
Tis	1 (0.9)	1 (2.9)	0	0
T0	12 (11.0)	0	0	12 (29.3)
T1	12 (11.0)	4 (11.4)	8 (24.2)	0
T2	22 (20.2)	2 (5.7)	9 (27.3)	11 (26.8)
T3	52 (47.7)	24 (68.6)	13 (39.4)	15 (36.6)
T4a	8 (7.3)	3 (8.6)	3 (9.1)	2 (4.9)
T4b	2 (1.8)	1 (2.9)	0	1 (2.4)
Pathological *N* stage, *n* (%)				
N0	79 (72.5)	22 (62.9)	21 (63.6)	36 (87.8)
N1a	13 (11.9)	5 (14.3)	6 (18.2)	2 (4.9)
N1b	5 (4.6)	1 (2.9)	2 (6.1)	2 (4.9)
N1c	6 (5.5)	4 (11.4)	1 (3.0)	1 (2.4)
N2a	4 (3.7)	2 (5.7)	2 (6.1)	0
N2b	2 (1.8)	1 (2.9)	1 (3.0)	0
Pathological TNM stage, *n* (%)				
0	12 (11.0)	1 (2.9)	0	11 (26.8)
I	27 (24.8)	5 (14.3)	12 (36.4)	10 (24.4)
II	38 (34.9)	16 (45.7)	9 (27.3)	13 (31.7)
III	28 (25.7)	12 (34.3)	12 (36.4)	4 (9.8)
IV	4 (3.7)	1 (2.9)	0	3 (7.3)
Positive/total harvested lymph nodes [median (range), *n*]	0 (0–22)/12 (1–51)	0 (0–14)/16 (3–33)	0 (0–22)/15 (5–51)	0 (0–2)/5 (1–23)
Positive/total central harvested lymph nodes [median (range), *n*]	0 (0–3)/3 (0–26)	0 (0–3)/4 (0–12)	0 (0)/4 (0–26)	0 (0)/2 (0–8)
Positive/total intermediate harvested lymph nodes [median (range), *n*]	0 (0–3)/3 (0–16)	0 (0–3)/4 (0–9)	0 (0–2)/4 (0–16)	0 (0)/2 (0–9)
Positive/total paraintestinal harvested lymph nodes [median (range), *n*]	0 (0–22)/4 (0–23)	0 (0–8)/7 (1–15)	0 (0–22)/6 (1–23)	0 (0–2)/2 (0–12)
Distance of distal resection margin [median (range), cm]	5 (1.0–10.0)	8 (5.0–10.0)	5 (1.0–10.0)	2.5 (1.0–10.0)
Positive resection distal margin, *n*	0	0	0	0

*TNM stage, tumor-node-metastasis classification; T stage, clinical tumor stage; N stage, clinical node stage*.

### Postoperative Outcomes

As shown in Table 4, the median length of stay (LOS) was 7 (range, 4–30) days. There was no postoperative mortality within 30 days. Eleven (10.1%) patients suffered complications after surgery, including 3 (2.8%) patients with anastomotic leakage, 1 (0.9%) patient with anastomotic bleeding, 1 (0.9%) patient with pelvic hemorrhage, 4 (3.7%) patients with intestinal obstruction, 2 (1.8%) patients with chylous leakage, and 1 (0.9%) patient with delayed wound healing. Only 5 (4.6%) and 8 (7.3%) patients developed urinary and sexual dysfunction, respectively. Details about the complication events are presented in Table 5. Among the patients who suffered postoperative complications, two patients required surgery, and nine patients received conservative treatment. All of these patients achieved recovery after invention.

**Table 4:** Postoperative and oncologic outcomes.

**Variables**	**Total (***n*** = 109 ,%)**	**Sigmoidectomy + HAR (***n*** = 48,%)**	**LAR (***n*** = 45,%)**	**APR (***n*** = 16,%)**
LOS after surgery [median (range), days]	7 (4–30)	7 (4–12)	7 (4–24)	8 (6–30)
30 day mortality	0	0	0	0
Postoperative complication	11 (10.1)	2 (4.2)	7 (15.6)	2 (12.5)
Anastomotic leakage	3 (2.8)	0	3 (6.7)	0
Anastomotic bleeding	1 (0.9)	1 (2.1)	0	0
Pelvic hemorrhage	1 (0.9)	0	1 (2.2)	0
Intestinal obstruction	4 (3.7)	0	3 (6.7)	1 (6.3)
Chylous leakage	2 (1.8)	1 (2.1)	1 (2.2)	0
Delay wound healing	1 (0.9)	0	0	1 (6.3)
Defecated dysfunction	38 (34.9)	9 (18.8)	29 (64.4)	0
Urinary dysfunction	5 (4.6)	0	1 (2.2)	4 (25.0)
Sexual dysfunction	8 (7.3)	0	4 (8.9)	4 (25.0)
Alive (NED)	102 (93.6)	45 (93.8)	41 (91.1)	16 (100)
Alive with tumor	6 (5.5)	2 (4.2)	4 (8.9)	0
Death due to tumor	1 (0.9)	1 (2.1)	0	0
Local recurrence	1 (0.9)	1 (2.1)	0	0
Distant metastasis	5 (4.6)	2 (4.2)	3 (6.7)	0

*HAR, high anterior resection; LAR, low anterior resection; APR, abdominoperineal resection; LOS, length of stay; NED, no evidence of disease*.

**Table 5:** Summary of postoperative complication events.

**Order**	**Gender**	**Age (years)**	**Tumor location**	**DAV (cm)**	**NACRT**	**Pathological stage**	**Types of operation**	**Complication**	**Complication detected on POD (days)**	**Invention**	**Invention outcome**	**LOS after surgery (day)**	**Survial status**
1	Female	37	Rectum	4	Yes	T2N0M0	LAR	Anastomotic leakage, Intestinal obstruction	2	Conservative treatment	Recovery	12	Alive (NED)
2	Male	59	Rectum	10	No	T3N1M0	LAR	Pelvic hemorrhage	5	Conservative treatment	Recovery	14	Alive (NED)
3	Male	47	Rectum	6	Yes	pCR	LAR	Intestinal obstruction	5	Conservative treatment	Recovery	12	Alive (NED)
4	Female	54	Rectum	7	No	T3N0M0	LAR	Intestinal obstruction	3	Conservative treatment	Recovery	12	Alive (NED)
5	Female	37	Sigmoid colon	25	No	T3N0M0	HAR	Chylous leakage	3	Conservative treatment	Recovery	10	Alive (NED)
6	Male	69	Rectum	5	Yes	T3N0M0	LAR	Chylous leakage	6	Conservative treatment	Recovery	9	Alive (NED)
7	Male	43	Rectum	3	Yes	pCR	APR	Intestinal obstruction	3	Operation	Recovery	24	Alive (NED)
8	Male	50	Rectum	1	Yes	T4N0M0	APR	Delay wound healing	8	Conservative treatment	Recovery	30	Alive (NED)
9	Male	68	Sigmoid colon	28	No	T3N2M0	Sigmoidectomy	Anastomotic bleeding	1	Conservative treatment	Recovery	10	Alive (NED)
10	Male	49	Rectum	10	No	T3N1M0	LAR	Anastomotic leakage	6	Conservative treatment	Recovery	24	Alive (NED)
11	Female	59	Rectum	6	Yes	T3N0M1	LAR	Anastomotic leakage	5	Operation	Recovery	11	Alive with tumor

*DAV, inferior tumor margin from the anal verge; NACRT, neoadjuvant chemoradiotherapy; POD, postoperative day; LOS, length of stay; pCR, pathological complete response; HAR, high anterior resection; LAR, low anterior resection; APR, abdominoperineal resection; NED, no evidence of disease*.

### Survival Analysis

**Figure 3:**
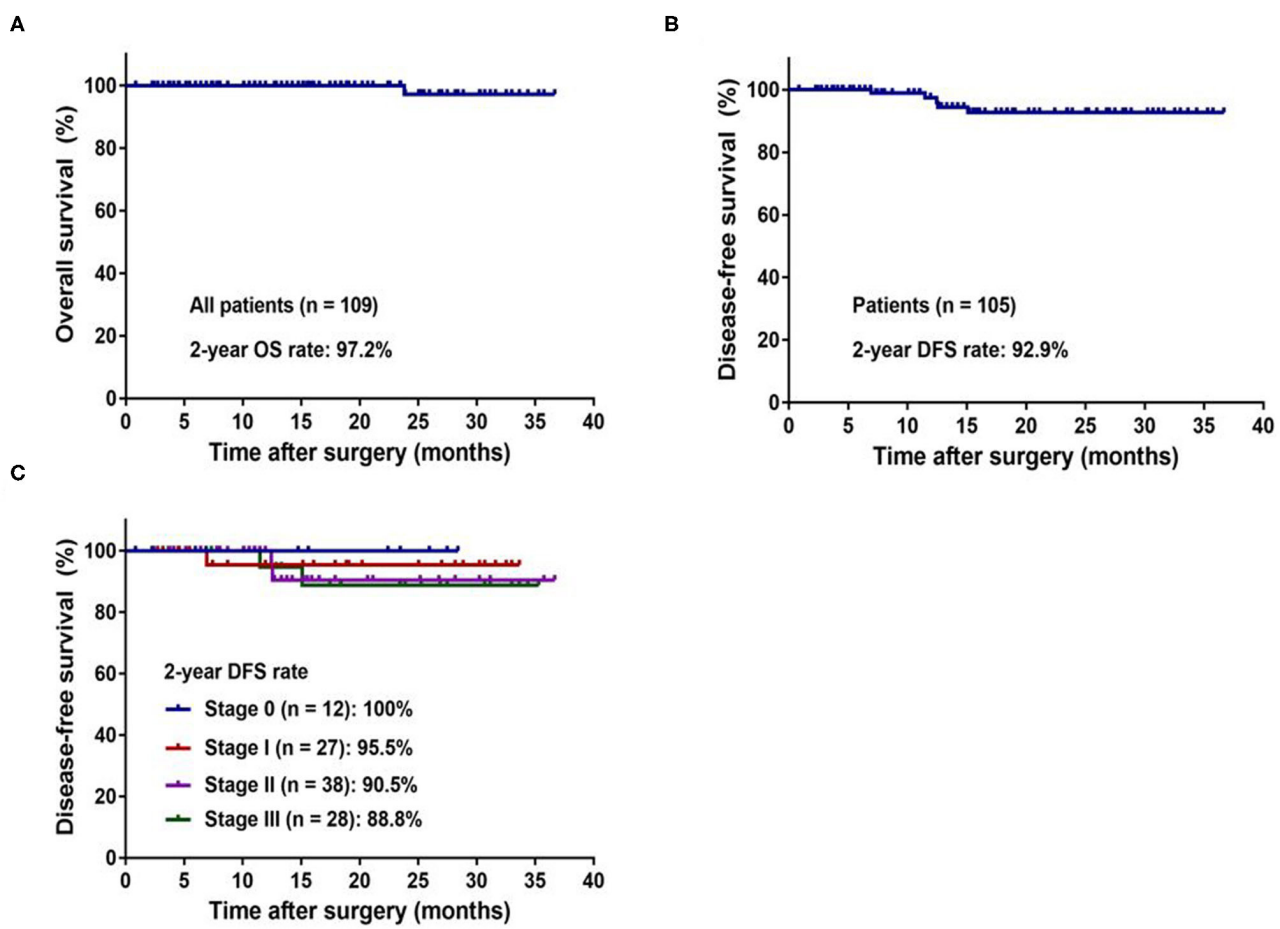
Kaplan–Meier curves of the patients with colorectal cancer underwent robotic surgery. **(A)** 2-year overall survival for whole study population. **(B)** 2-year disease-free survival for non-metastatic patients. **(C)** 2-year disease-free survival by pathologic stage.

The median follow-up period for all patients was 17 months (range 1–37 months). One hundred and two patients (93.6%) in our study cohort were alive with no evidence of disease. One (0.9%) patient developed local recurrence, and 5 (4.6) patients developed distant metastasis. One patient died due to disease progression. The 2-year OS rate of all patients (*n* = 109) was 97.2% (Figure 3A), and the 2-year DFS rate of non-metastatic patients (*n* = 104) was 92.9% (Figure 3B). The 2-year DFS rate of patients in stages 0, I, II, and III were 100, 95.5, 90.5, and 88.8%, respectively (Figure 3C).

## Discussion

In this retrospective study, we investigated the surgical and oncological outcomes of robotic resection for sigmoid and rectal cancer in Chinese patients. Our data found that robotic surgery had a low conversion rate, low morbidity rate, and remarkable oncological outcomes, which confirms its safety and feasibility in Chinese patients with sigmoid and rectal cancer.

Rectal cancer resection is very difficult to perform using traditional laparotomy, but laparoscopic surgery has an advantage for rectal surgery under a clearer view despite the narrow and deep pelvic space. Several studies (12–14, 16, 17) confirmed that laparoscopic surgery presented better short-term outcomes and comparable long-term outcomes compared to traditional laparotomy. The surgical advantages and comparable oncological outcomes of laparoscopic surgery were clearly demonstrated in patients with locally advanced rectal cancer after preoperative chemoradiotherapy in the COREAN trial (18). Because of the features of robotic technology, robotic surgery is much more advantageous, especially for patients with locally advanced rectal cancer after treatment with preoperative chemoradiotherapy. In our study, 37.6% patients presented with a BMI ≥ 24 kg/m^2^, and 55.4% patients with rectal cancer received neoadjuvant treatment. No conversion occurred with a median procedure time of 270 min, a median estimated blood loss of 50 ml and a median length of stay of 7 days. Only 11 patients (10.1%) experienced postoperative complications, which shows the remarkable surgical advantages of robotic surgery in patients with rectal cancer who received neoadjuvant treatment.

As previously reported, the most commonly encountered complication was anastomotic leakage, and its average occurrence rate was 8.6% (range from 1.2 to 20.5%) (19, 20) and 1.8 to 13.6% in robotic surgery (21, 22). Its occurrence affects the patient's quality of life, increases hospitalization costs, delays the implementation of adjuvant chemotherapy, and shortens the overall survival (22, 23). Eleven patients (10.1%) had postoperative complications, which included 3 patients who suffered anastomotic leakage. Due to the advantages of robotic surgery, such as 3D magnified view, wristed instruments and stable camera platform, surgeons are able to maintain the sufficient surgical dissection plane down to the pelvic floor, which minimizes damage to marginal vessels and allows performance of the rectal division and reconstruction efficiently and safely to shorten the procedure time.

More precise surgery also helps protect the autonomic nerves and reduce the occurrence of long-term postoperative complications, including defecation, urinary and sexual dysfunction (14). Wang and coworkers (24) described a significant increase in International Prostate Symptom Score (IPSS) after surgery in the laparoscopic group, and more patients in the laparoscopic group (34.8%) perceived a severe damage in their overall level of sexual function following surgery than the patients in the robotic group (18.3%). Several studies (25, 26) also claimed that robotic TME improved the preservation of urinary and sexual functions because the arms of the robotic device are stable and highly flexible in the separation and exposure of tissues. With the high-resolution lens of the da Vinci surgical system to effectively recognize the nerve, the application of the PANP technique resulted in a significant reduction in the incidence of urinary dysfunction (4.6%) and sexual dysfunction (7.3%) in our study.

A positive circumferential margin or insufficient harvested lymph nodes leads to local recurrence (27). Although the relationship between sufficiently harvested lymph nodes and local recurrence rate is controversial, the guidelines list the harvesting of < 12 lymph nodes as risk factor and noted that the performance of TME with clear surgical margins and adequate lymph node dissection were related to lower recurrence rate (28, 29). In our study, the median positive total harvested lymph nodes was 0 (range 0–22), and the total harvested lymph nodes was 12 (range 1–51). The 2-year DFS of patients in stages 0, I, II, and III were 100, 95.5, 90.5, and 88.8%, respectively, and the 2-year DFS of patients in stage III was slightly better than previous studies (65.2–82.8%) (12, 13, 17). The high quality of the procedure (no positive resection distal margin and sufficient harvested lymph nodes) and neoadjuvant treatment contributed to the remarkable oncological outcomes.

Although routine mobilization of the splenic flexure is not necessary during anterior resection for rectal cancer, it is one of the important surgical step in some of sigmoid and rectal cancer resection, which aimed to ensure a tension-free with good blood supply (30, 31). However, splenic flexure mobilization was recognized as a challenging step for robotic surgery. It was well-known that the splenic flexure anatomy is complex, which consisted of multiple vessels, surrounding vulnerable organs, such as spleen, and irregular adhesions. In addition, this step would be usually more difficult due to the lack of operating space (32). Moreover, due to limited range of motion of the robotic arms and surgical field compromising multiple quadrants, mobilization of the splenic flexure required a series of procedure, including removing robotic arms, replacing the patient cart and even reconnecting the robot system (33, 34). Therefore, when progress was difficult, we would mobilize splenic flexure by using laparoscopy approach in our center. Since there was no such case receiving splenic flexure mobilization, we were unable to provided any technical skills and surgical outcome of this step in the current study.

Several limitations should be acknowledged in the present study. First, this retrospective descriptive study included an uncontrolled, single-arm methodology and a limited number of patients from a single cohort. Although our study confirms the safety and feasibility of robotic surgery in Chinese CRC patients, the findings must be validated in a prospective, multicenter clinical trial with a large population in the future. Second, the short follow-up duration was insufficient to evaluate 5-year survival outcomes, which may have led to a misestimation of the effect of robotic surgery on OS and DFS. Considering the short follow up mean time, oncological results are derived from the pathological specimen anlaysis, that indirectely might confirm good survival rate. Additionally, selective bias undeniably exists in our cohort.

## Conclusion

Robotic surgery is technically feasible and safe for Chinese CRC patients, especially for rectal cancer patients receiving neoadjuvant treatment because a robotic laparoscope with large magnification shows a clear surgical space for tumor resection in cases of mesorectal edema. Due to the advantages of robotic surgery, surgeons are able to perform the procedure efficiently and safely and help protect marginal vessels and the autonomic nerves, which reduces the occurrence of short-term and long-term postoperative complications and ensures clear surgical margins and adequate lymph node dissection.

## Data Availability Statement

The datasets used and analyzed during the current study are available from the corresponding author on reasonable request. The authenticity of this article has been validated by uploading the key raw data onto the Research Data Deposit public platform (http://www.researchdata.org.cn), with the Approval Number as RDDA2021002030.

## Ethics Statement

The present study was performed according to the ethical standards of the World Medical Association Declaration of Helsinki and was approved by the Institutional Review Board and Independent Ethics Committees of Sun Yat-sen University Cancer Center. The informed consent requirement was waived by the ethics committees based on the nature of this retrospective study, in which patient data were kept confidential.

## Author Contributions

JP and WL analyzed and interpreted the data. JL and ZP were the chief surgeons who performed the surgery, the chemotherapy, and all authors participated. JP, WL, JL, and ZP were major contributors in writing the manuscript. All authors read and approved the final manuscript.

## Conflict of Interest

The authors declare that the research was conducted in the absence of any commercial or financial relationships that could be construed as a potential conflict of interest.

## Publisher's Note

All claims expressed in this article are solely those of the authors and do not necessarily represent those of their affiliated organizations, or those of the publisher, the editors and the reviewers. Any product that may be evaluated in this article, or claim that may be made by its manufacturer, is not guaranteed or endorsed by the publisher.
